# Activation of intestinal olfactory receptor stimulates glucagon-like peptide-1 secretion in enteroendocrine cells and attenuates hyperglycemia in type 2 diabetic mice

**DOI:** 10.1038/s41598-017-14086-5

**Published:** 2017-10-25

**Authors:** Ki-Suk Kim, In-Seung Lee, Kang-Hoon Kim, Jiyoung Park, Yumi Kim, Jeong-Hee Choi, Jin-Sung Choi, Hyeung-Jin Jang

**Affiliations:** 1 Department of Biochemistry, College of Korean Medicine, Kyung Hee University, 26, Kyungheedae-ro, Dongdaemun-gu, Seoul, 02447 Republic of Korea; 2 College of Pharmacy, The Catholic University of Korea, Bucheon, Gyeonggi-do 14662 South Korea

## Abstract

Odorants are non-nutrients. However, they exist abundantly in foods, wines, and teas, and thus can be ingested along with the other nutrients during a meal. Here, we have focused on the chemical-recognition ability of these ORs and hypothesized that the odorants ingested during a meal may play a physiological role by activating the gut-expressed ORs. Using a human-derived enteroendocrine L cell line, we discovered the geraniol- and citronellal-mediated stimulation of glucagon-like peptide-1 (GLP-1) secretion and elucidated the corresponding cellular downstream signaling pathways. The geraniol-stimulated GLP-1 secretion event in the enteroendocrine cell line was mediated by the olfactory-type G protein, the activation of adenylyl cyclase, increased intracellular cAMP levels, and extracellular calcium influx. TaqMan qPCR demonstrated that two ORs corresponding to geraniol and citronellal were expressed in the human enteroendocrine cell line and in mouse intestinal specimen. In a type 2 diabetes mellitus mouse model (*db/db*), oral administration of geraniol improved glucose homeostasis by increasing plasma GLP-1 and insulin levels. This insulinotropic action of geraniol was GLP-1 receptor-mediated, and also was glucose-dependent. This study demonstrates that odor compounds can be recognized by gut-expressed ORs during meal ingestion and therefore, participate in the glucose homeostasis by inducing the secretion of gut-peptides.

## Introduction

Odorants, which are aromatic compounds, are abundant in foodstuffs and make up the flavor of foods as well as the taste. For example, eugenol abundantly exists in cloves; benzaldehyde represents the smell of bitter almond, and 3-isobutyl-2-methoxypyrazine (IBMP) causes the distinctive flavor of Cabernet Sauvignon^1–3^.

Distinguishing individual flavors between each food item depends on the recognition of the taste and odor compounds through the corresponding taste and olfactory receptors (ORs). Despite only one receptor existing for each sweet and umami taste and 25 bitter taste receptor genes being expressed, humans can distinguish numerous flavors, as ~350–400 ORs are expressed in the human olfactory epithelium (OE) for olfactory sensation^4^. These ORs are able to sense single or multiple odorants, and a single odorant can be recognized by multiple receptors^5^. This feature shows the limitlessness of the flavor recognition ability of ORs. OR genes, which make up 3% of the human genome, are expressed not only in the nose but also at in various non-chemosensory organs, including the gastrointestinal (GI) tract^6^. The functions of these gut-expressed ORs have not yet been investigated.

We previously investigated taste receptors expressed in the gut and their activation via tastant binding, which leads to the secretion of glucagon-like peptide-1 (GLP-1), an incretin hormone that induces a decrease in blood glucose levels by stimulating insulin secretion^7–9^. We also found that the mRNA signals for several ORs in human enteroendocrine NCI-H716 cells are upregulated by the bitter tastant quinine as well as herbal extracts^10–12^. Therefore, we hypothesized that some ORs and their signaling pathways are involved in GLP-1 secretion in enteroendocrine cells and thus are able to participate in glucose homeostasis.

In the OE, olfaction is mediated by the OR, a seven transmembrane G protein-coupled receptor (GPCR). When the odorant binds to its receptor, cellular downstream signaling is initiated through the activation of the olfactory-type G protein (G_olf_), which activates adenylyl cyclase (AC) to increase intracellular cAMP levels^13^. This activation of the cAMP signaling pathway by odorant stimulation results in an influx of Ca^2+^ through the cyclic nucleotide-gated (CNG) channel A2 and subsequent depolarization^14^. We hypothesized that gut olfaction also occurs through similar cellular downstream signaling events as those in the OR signaling cascade for the nose.

## Results

**Figure 1:**
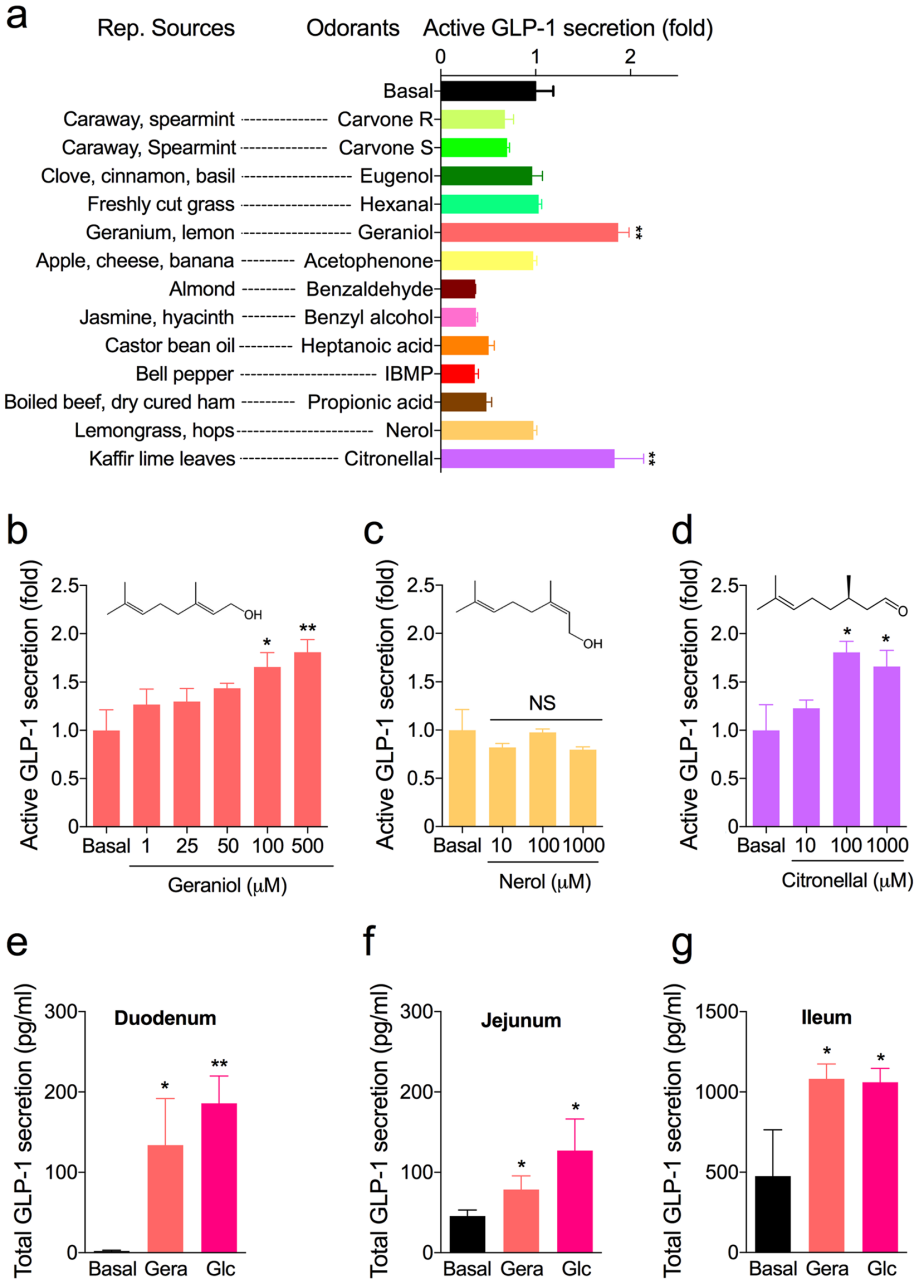
Odorant-stimulated GLP-1 secretion. (**a**) Active GLP-1 secreting effects of different odorants were compared to the effect of vehicle (Basal) on human enteroendocrine NCI-H716 cells. The representative sources of correspond to each odorant are indicated. (**b**–**d**) Active GLP-1 secretions in NCI-H716 cells respond to geraniol (**b**) and citronellal (**d**), but not nerol (**c**). (**e**–**g**) Total GLP-1 secretions in intact mouse intestinal tissues: duodenum (**e**), jejunum (**f**), and ileum (**g**), respond to geraniol (Gera;100 μM) and glucose (Glc; 10% of media volume), respectively. Error bars show SEM. Statistics, ANOVA followed by Dunnett’s post-hoc. *p < 0.05, **p < 0.01. ^#^NS, not significant; IBMP, isobutyl-3-methoxypyrazine.

To demonstrate that olfaction in the gut is related to an endocrine event, we measured the GLP-1-stimulating efficacy of various odorants using the endocrine-differentiated NCI-H716 cells. NCI-H716 cell line is a poorly differentiated adenocarcinoma derived from a human cecal fluid^15^. Culturing the cells on the matrigel-precoated culture dishes causes the cells to exhibit the properties of enteroendocrine L cell, including their chromogranin and proglucagon expressions^15,16^. NCI-H716 cells secrete GLP-1 in response to a number of neurotransmitters, nutrients and secretagogues, as described in numerous studies^7–9,16^ In the endocrine-differentiated NCI-H716 cells, geraniol, a monoterpene alcohol abundant in geraniums, grapes, lemons, and roses, exhibited the most significant GLP-1 stimulatory effect (Fig. 1a). The chemical structures of each odorant are provided online (Supplementary Fig. S1). For doses of 100- to 500 μM, geraniol significantly induced GLP-1 secretion in NCI-H716 cells in a dose-dependent manner (Fig. 1b). Nerol, which is an isomer of geraniol, had no effect on GLP-1 secretion in NCI-H716 cells (Fig. 1c). We also found that citronellal, which activates the same ORs activated by geraniol^17^, stimulated GLP-1 secretion as well (Fig. 1a,d). Both the geraniol and citronellal at a dose of 100 μM showed similar GLP-1 secreting efficacy levels (1.7-fold relative to the basal level). Geraniol had no effect on cell viability at any dose (Supplementary Fig. S2).

To determine if the results from the NCI-H716 cells have any direct bearing on intestinal L cells *in situ*, we isolated mouse intestinal tissues from the duodenum, jejunum, and ileum and measured GLP-1 secretion in response to a treatment of geraniol or glucose. There was a significant increase in GLP-1 release from each of the mouse intestinal tissues after the addition of 100 μM geraniol or 10% glucose (Fig. 1e–g). We observed nearly 10-times greater GLP-1 secretion from the mouse ileum (Fig. 1g) as compared to the duodenum and jejunum (Fig. 1e,f) even after a saline treatment (basal).

**Figure 2:**
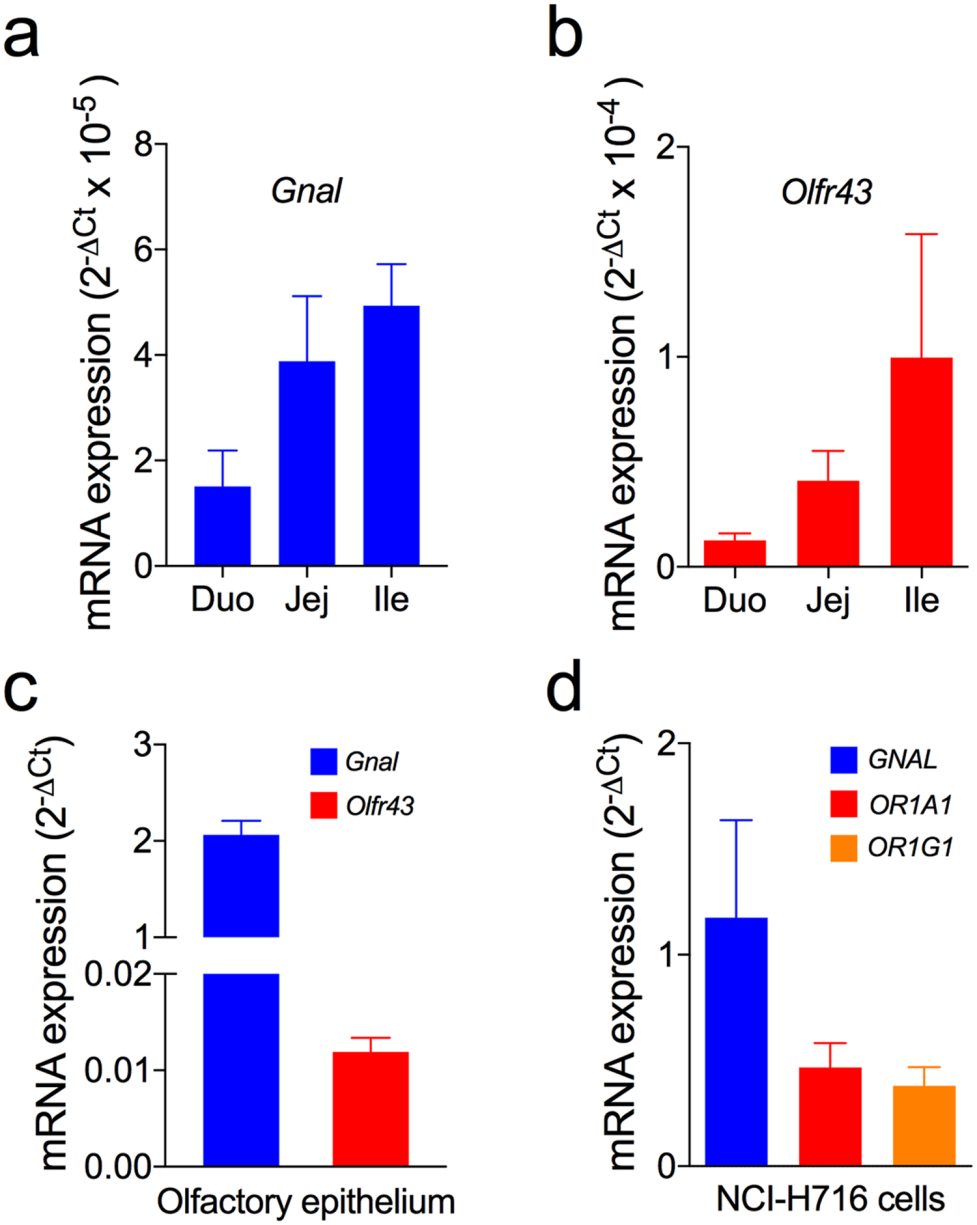
Olfactory receptor expression in enteroendocrine L cells. The Taqman gene expression assay demonstrated the relative *Gnal* (**a**) and *Olf43* (**b**) gene expression levels in mouse duodenum (Duo), jejunum (Jej), and ileum (Ile) specimens. (**c**) The relative *Gnal* and *Olf43* gene expression levels in mouse olfactory epithelium. (**d**) Relative *GNAL*, *OR1A1*, and *OR1G1* gene expression levels in the differentiated NCI-H716 cells.

Subsequently, we attempted to determine whether the ORs corresponding to geraniol and citronellal are expressed in the mouse intestinal tissues and in NCI-H716 cells. TaqMan qPCR was used to determine the mRNA expression levels of *Gnal*, a mouse olfactory G protein gene, and *Olfr43*, which is homologous to the human *OR1A1* gene^18^, in mouse intestinal specimens (Fig. 2a,b). The mRNA expression levels of *Gnal* and *Olfr43* in the mouse intestinal tissues are much lower than the levels in the mouse olfactory epithelium (Fig. 2c). The mRNAs for *GNAL* (human G_olf_ gene), *OR1A1*, and *OR1G1* are also expressed in differentiated NCI-H716 cells (Fig. 2d). Mock controls without reverse transcriptase, performed for *GNAL*, *OR1A1*, and *OR1G1*, yielded no amplification for the NCI-H716 cells.

**Figure 3:**
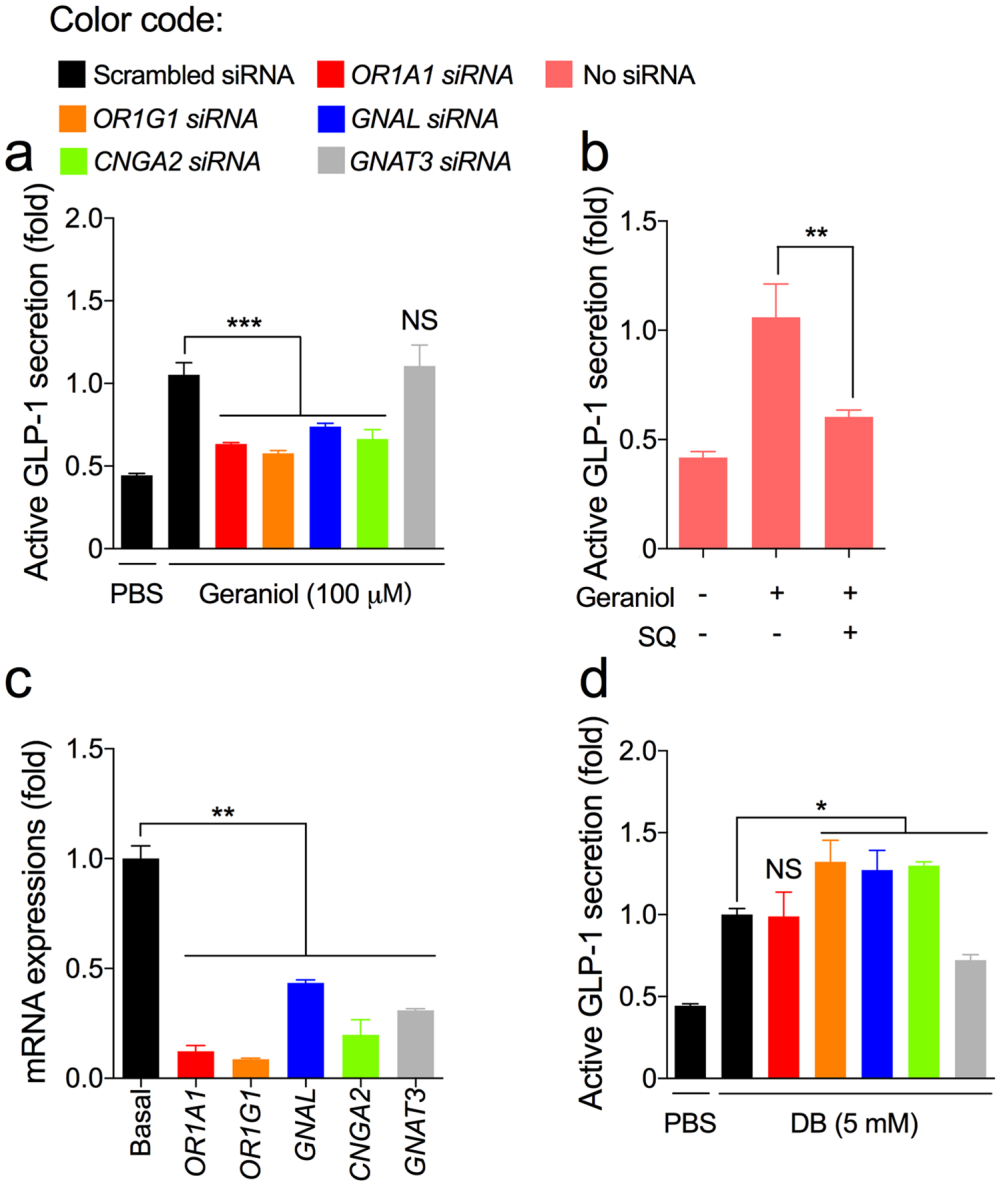
Downstream signaling of geraniol-mediated GLP-1 secretion. (**a**) The active GLP-1-secreting effect of geraniol (100 μM) was blocked by transfections with siRNAs for the olfactory receptor genes, *OR1A1* and *OR1G1*, *CNGA2*, and the olfactory G protein *α*-subunit gene, *GNAL*, but not for the taste G protein *α*-subunit gene, *GNAT3*, in differentiated NCI-H716 cells. (**b**) An AC inhibitor SQ22536 (SQ; 50 μM) blocked the GLP-1 secretion-inducing activity of geraniol. (**c**) Transfection of each siRNA successfully downregulated the corresponding target mRNA expression levels. (**d**) Olfactory siRNA transfection did not reduce the GLP-1-secreting effect of the bitter tastant denatonium benzoate (DB). Error bars show SEM. Statistics ANOVA followed by Dunnett’s post-hoc. *p < 0.05, **p < 0.01, ***p < 0.001. # NS, non-significant.

We determined the mode-of-action underlying the GLP-1 stimulatory effect of geraniol in the NCI-H716 cells. Transfection with the siRNAs of either *OR1A1* or *OR1G1* inhibited the GLP-1 secretion-inducing activity of geraniol (Fig. 3a). GLP-1 secretion was also inhibited by transfection with the *GNAL* and *CNGA2* siRNAs or by a treatment with the AC inhibitor SQ22536 (Fig. 3a,b). The inhibition of *GNAT3*, which encodes the taste G protein Gα-gustducin, did not affect the GLP-1 secretion-inducing activity of geraniol (Fig. 3a). Each of the mRNA expression levels in the endocrine-differentiated NCI-H716 cells were successfully downregulated after corresponding siRNA transfections (Fig. 3c). To validate the siRNA-transfection results, we performed the same GLP-1 secreting study using the bitter tastant denatonium benzoate (DB), which stimulates GLP-1 secretion through the activation of Gα-gustducin^8^. *OR1A1* siRNA transfection did not affect the GLP-1-secreting effect of DB, whereas transfection with *GNAT3* siRNA significantly decreased GLP-1 secretion (Fig. 3c). Interestingly, *OR1G1*, *GNAL*, and *CNGA2* siRNA transfections increased GLP-1 secretion in response to a DB treatment (Fig. 3c).

**Figure 4:**
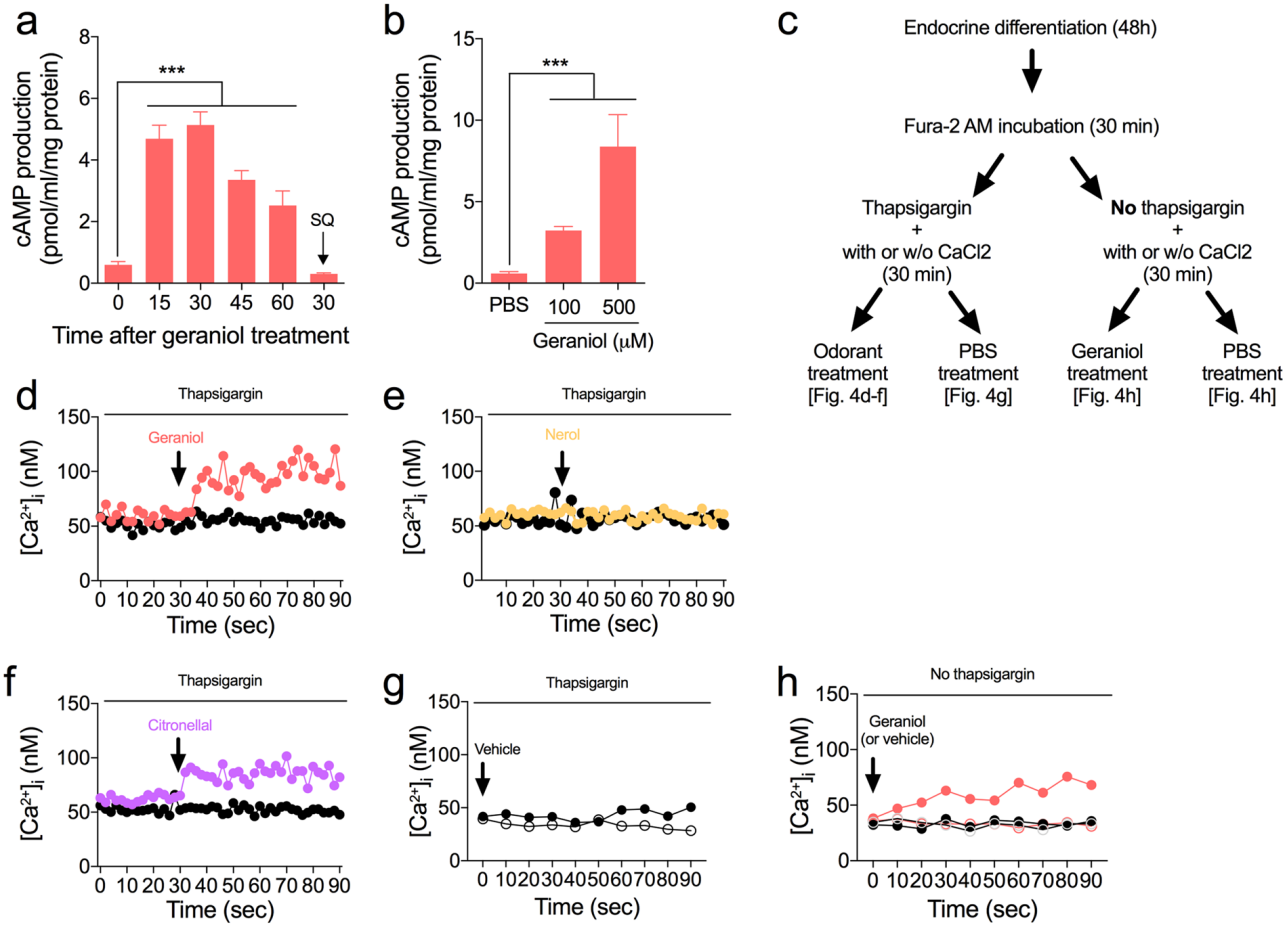
Intracellular second messengers increased by odorant treatment. (**a**) Time-dependent cAMP increasing effect of geraniol treatment was abolished in the AC inhibitor SQ22536 (SQ; 50 μM)-treated NCI-H716 cells. (**b**) Dose-dependent cAMP production in response to geraniol. Error bars show SEM. Statistics ANOVA followed by Dunnett’s post-hoc. ***p < 0.001. (**c**–**h**) Odorant-induced extracellular calcium influx into the NCI-H716 cells. (**c**) Schematic overview of the series of experiments was provided. Cells were pre-treated with thapsigargin to induce depletion of intracellular calcium store before stimulations. Cells were maintained under two different conditions, presence (filled circles) or absence (blanked circles) of extracellular calcium. Geraniol (**d**) and citronellal (**f**), but nerol (**e**) induced extracellular calcium influx into the thapsigargin-treated NCI-H716 cells. Black arrow indicates each odorant (100 μM) or vehicle stimuli. (**g**–**h**) Vehicle treatment did not affect the intracellular calcium levels regardless the exist of extracellular calcium in both in presence (**g**) or absence (**h**) of thapsigargin. (**h**) Geraniol stimulated [Ca^2+^]_i_ release without thapsigargin pretreatment in the absence of extracellular calcium. Graph indicates only 15 out of 32 cells those were respond to the geraniol treatment.

We also measured the geraniol-provoked cAMP production in NCI-H716 cells. The production of cAMP, an intracellular second messenger, stimulated by geraniol (100 μM) was initially observed at 15 min after the treatment, reaching the highest concentration (approximately 5-fold to the basal level) at 30 min (Fig. 4a). Treatment with the AC inhibitor SQ22536 blocked the intracellular cAMP-producing effect of geraniol (Fig. 4a). Additionally, we observed dose-dependent cAMP production by a geraniol treatment at 30 min after the treatment (Fig. 4b).

We also investigated odorant-stimulated increases in the intracellular calcium concentration [Ca^2+^]_i_ in NCI-H716 cells, as would be expected with the extracellular calcium influx upon stimulation. We depleted the intracellular calcium store by treating the cells with thapsigargin, a non-competitive and an irreversible inhibitor of the sarco/endoplasmic reticulum Ca^2+^ ATPase (SERCA)^19^, before stimulation with the odorants. Thapsigargin treatment is known to be results in an immediate increase of [Ca^2+^]_i_ due to the depletion of the intracellular calcium store (*i.e*., endoplasmic reticulum), and subsequently resulting in a stable plateau within 3 minutes^20^. The subsequent addition of extracellular calcium, such as a CaCl_2_ solution, produces a sustained intracellular calcium influx^21^. Given that OR activation in the olfactory epithelium is accompanied by an extracellular calcium influx by the CNGA2 channel^14^, we hypothesized that geraniol and citronellal will accelerates the extracellular calcium influx at this stage. A schematic overview of the experiments is provided (Fig. 4c). Upon geraniol treatment, [Ca^2+^]_i_ increased in cells with extracellular calcium while there was no effect on [Ca^2+^]_i_ in cells without extracellular calcium, indicating that geraniol caused an extracellular calcium influx into the NCI-H716 cells (Fig. 4d). Nerol treatment had no effect on [Ca^2+^]_i_, in cells with or without extracellular calcium (Fig. 4e). The results with citronellal were similar to those with geraniol (Fig. 4f). Vehicle treatment had no effects on [Ca^2+^]_i_, in cells (Fig. 4g,h). The geraniol treatment also resulted in an increase of [Ca^2+^]_i_ without a thapsigargin treatment, indicating CNGA2-independent activity of geraniol in the NCI-H716 cells; however, the effect was moderate, and only 15 out of 32 observed cells were responded to the stimulation. (Fig. 4h).

**Figure 5:**
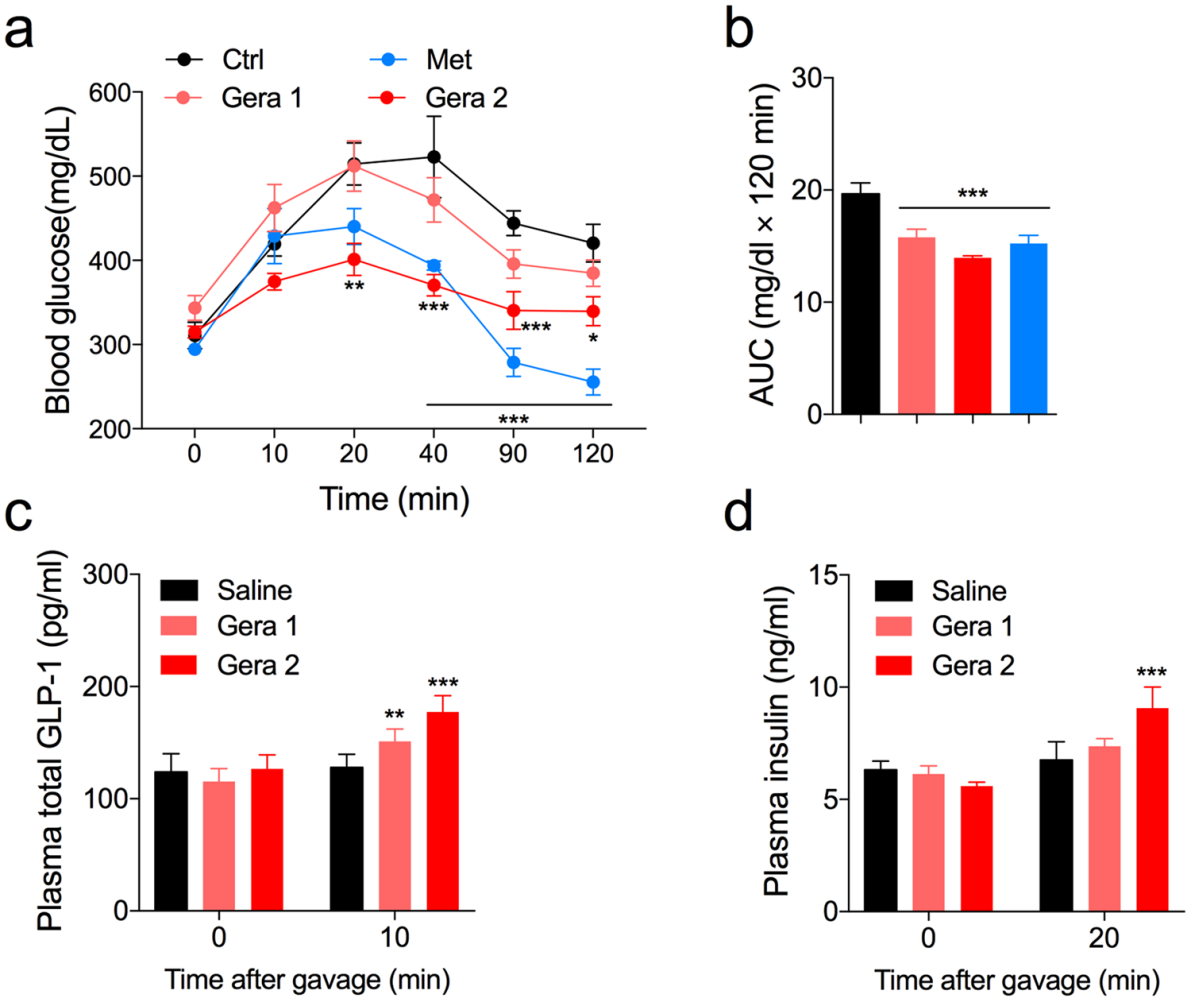
Geraniol regulates blood glucose levels by increasing plasma total GLP-1 and plasma insulin secretion in *db/db* mice. (**a**) Blood glucose lowering effects of geraniol (Gera 1, geraniol 1 mmol/kg; Gera 2, geraniol 2 mmol/kg) oral administration in the *db/db* mice were compared to the saline- or metformin (Met 2.3 mmol/kg) oral administration during the OGTT (5 g/kg). *n* = 6-7. (**b**) AUC (120 min) of blood glucose after glucose gavage. (**c**) Plasma total GLP-1 increased by geraniol treatments in the *db/db* mice after glucose gavage (2 g/kg). (**d**) Plasma insulin increased by geraniol treatments in the *db/db* mice after glucose gavage (2 g/kg). *n* = 6. Error bars show SEM. Statistics, ANOVA followed by Tukey’s post hoc *p < 0.05, **p < 0.01, ***p < 0.001.

We then hypothesized that geraniol might also stimulate the secretion of GLP-1 from enteroendocrine L cells in an *in vivo* model. We performed an OGTT in *db/db* mice, which show the fasting blood glucose levels ranged from 300 to 400 mg/dl. Geraniol (1- and 2 mmol/kg) resulted in decreased blood glucose levels compared with those in the saline-treated group (Fig. 5a,b). The blood glucose lowering effect of geraniol was compared to the effect of metformin (2.3 mmol/kg). A low dose of geraniol (1 mmol/kg) decreased the AUC by 15% during OGTT while a high dose (2 mmol/kg) decreased it by 25% (Fig. 5b).

During the OGTT, geraniol resulted in increased plasma total GLP-1 (Fig. 5c) and plasma insulin levels (Fig. 5d). Geraniol at less than 1 mmol/kg (0.2 mmol/kg and 0.7 mmol/kg) had no effect on the blood glucose levels of *db/db* mice during the OGTT (Supplementary Fig. S3).

**Figure 6:**
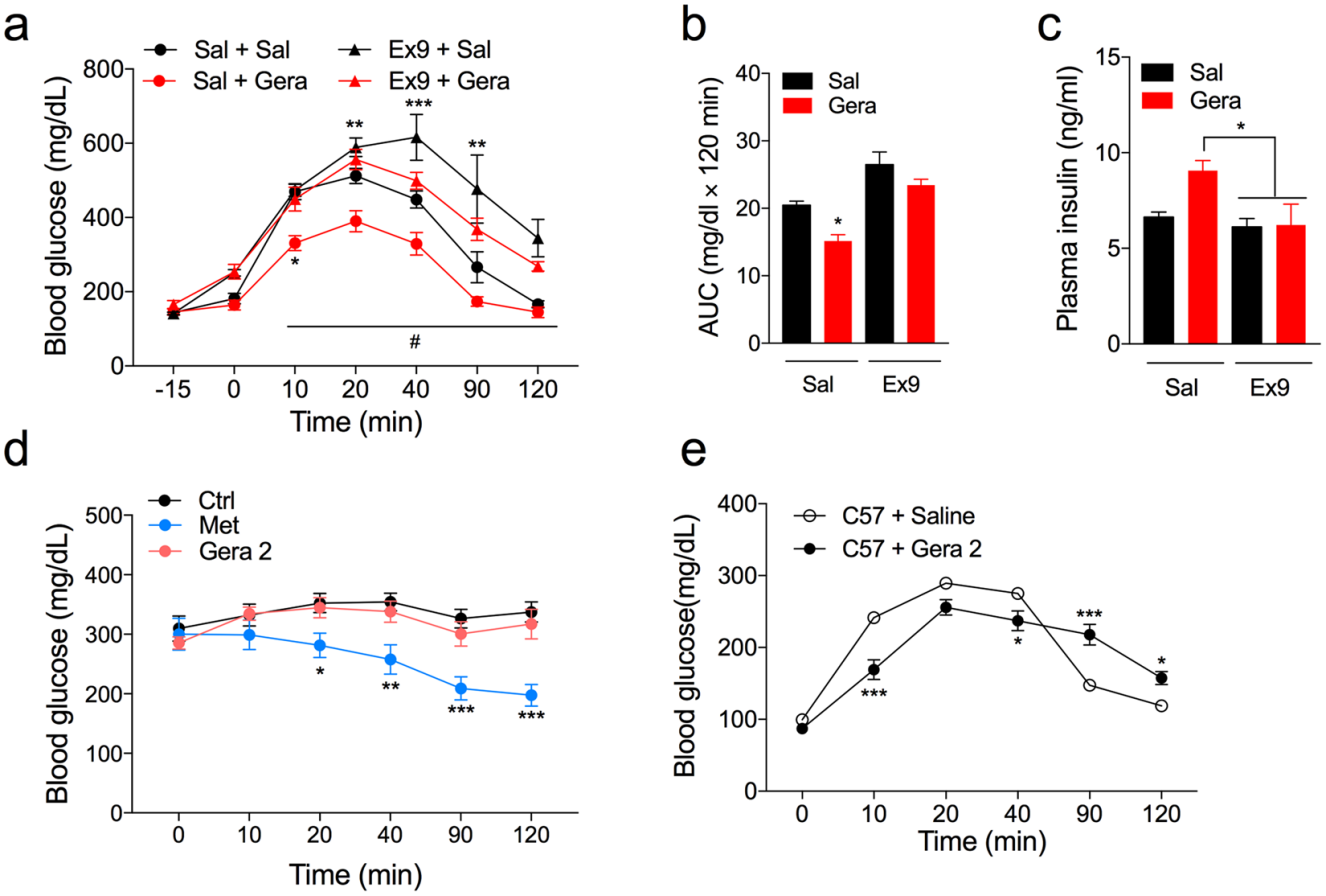
The incretin effect of geraniol treatment in *db/db* mice. (**a**–**c**) In *db/db* mice, IP injection of Ex9 or saline were performed 15 min prior to the glucose gavage (5 g/kg). Geraniol (2 mmol/kg) or saline were orally administrated just before the glucose gavage. Geraniol administration did not affect the blood glucose (**a**) and plasma insulin levels (**c**) in Ex9 pre-injected *db/db* mice. *n* = 5. (**b**) AUC (120 min) of blood glucose after glucose gavage. (**d**) Geraniol did not affect the blood glucose levels in *db/db* mice without glucose gavage while metformin decreased the blood glucose levels. (**e**) Geraniol administration retarded, but not improved glucose homeostasis during OGTT in non-diabetic C57BL/6 mice. Error bars show SEM. Statistics, ANOVA followed by Tukey’s post hoc. *p < 0.05, **p < 0.01, ***p < 0.001.

To examine whether the blood glucose lowering effect of geraniol is due to its GLP-1 secreting effect and the consecutive insulin secretion, we intraperitoneally injected Ex9-39 (Ex9), a potent GLP-1 receptor antagonist, into the *db/db* mice and performed OGTT after a geraniol treatment. Ex9 reduces insulin secretion following the blockade of GLP-1 action *in vivo* ^22,23^. We observed that an Ex9 pre-injection increased blood glucose levels of *db/db* mice with a saline treatment at 20 min after OGTT (Fig. 6a). In Ex9 pre-injected *db/db* mice, geraniol (2 mmol/kg) failed to lower blood glucose levels during the OGTT, while it dramatically lowered blood glucose levels in saline pre-injected *db/db* mice (Fig. 6a,b). A geraniol treatment did not affect the plasma insulin levels of Ex9 pre-injected mice after 20 min of glucose gavage (Fig. 6c). Geraniol oral administration did not affect the blood glucose levels of *db/db* mice in the absence of glucose gavage, while metformin induced hypoglycemia (Fig. 6d). In C57BL/6 mice, a geraniol treatment resulted in a slow increase of blood glucose levels during 40 min after glucose gavage and also in a slow return of the increased blood glucose to the basal level (Fig. 6d).

## Discussion

Olfaction, or odorant sensing, is a receptor-mediated chemical perception process that occurs in the nasal cavity. However, OR expressions have been found not only in the nose, but also in various non-chemosensory organs, including the testis, heart, liver, kidney, brain, muscle, lung, and the GI tract^6,24–28^. The functions of these ORs in non-chemosensory organs have not yet been investigated, but Kidd *et al*., who determined that certain odorants stimulate serotonin secretion in human enterochromaffin cells, suggested that gut-expressed ORs might be involved in a physiological event^29^. The expression of ORs occurs in isolated human enterochromaffin cells^6^ and is responsive to single or multiple odorants^5^.

We screened for the GLP-1 secreting effect of 13-odorants in enteroendocrine NCI-H716 cells and found that geraniol- and citronellal-stimulated GLP-1 secretion. There are structural similarities between these two odorants; both are a 10-carbon monoterpenoid with branches at C2 and C6. These structural characteristics may provide the odorants with a binding motif for OR1A1 and OR1G1^17,30^. The mRNA expressions of the ORs and their intracellular signals in the human enteroendocrine L cell line and mouse intestinal tissues support the hypothesis that the activation of intestinal ORs may be involved in physiological events, *i*.*e*., GLP-1 secretion. These findings demonstrate that intestinal L cells are able to detect odor compounds during meal ingestion like olfactory cells do in the OE and also that the intestinal odorant sensation is able to stimulates GLP-1 secretion, which subsequently regulates glucose homeostasis.

Geraniol-stimulated GLP-1 secretion in NCI-H716 cells was associated with the OR signaling pathway, which involves *GNAL* and *CNGA2* and AC activation. Unlike the other taste receptor-mediated GLP-1 secreting events in the enteroendocrine cells, which activate the inositol triphosphate (IP_3_)-mediated intracellular calcium store, odor-stimulated GLP-1 secretion mainly recruits calcium ions from an extracellular source. Several studies have reported that geraniol perception by OE is largely absent in CNGA2-KO mice and AC type 3 (AC3)-KO mice^14,31^. This lends support to the idea that the activation of ORs results in CNGA2-mediated calcium influx to induce the depolarization of NCI-H716 cells. Indeed, geraniol- and citronellal resulted in a calcium influx from an extracellular source into enteroendocrine cells when we depleted the intracellular calcium store to prevent the IP_3_-mediated [Ca^2+^]_i_ increase. The provoked cAMP- and the consecutive influx of the extracellular calcium-mediated signaling pathway of the ORs in the enteroendocrine L cells are similar to these processes by olfactory neurons. However, instead of causing a neurotransmitter release, enteroendocrine L cells secrete GLP-1 after OR activation.

Although the effect was moderate, and given that not all of the cells responded to the stimuli, geraniol also was able to invoke [Ca^2+^]_i_ without a thapsigargin pretreatment, indicating IP_3_-mediated [Ca^2+^]_i_ release is also available in the NCI-H716 cells. This extracellular calcium-independent activity of the geraniol on the release of [Ca^2+^]_i_ may be mediated by the Gα16 protein. One study^30^ have reported that 10–15% of OR1G1/Gα16-expressing HEK293 cells were able to respond to a geraniol treatment (10uM). Whether this Gα16-mediated [Ca^2+^]_i_ release is physiologically relevant is unknown.

OR genes occupy a considerable amount (3%) of human genome. Humans express approximately ~400 ORs, and some expressed in enteroendocrine cells are able to participate in postprandial gut-peptide secretion during a meal. Though the GLP-1 secreting efficacy of a single odorant is moderate (>2-fold), considering how many odorants are consumed during a meal, the odorant-stimulated gut-peptide secretion event might be more important than previously considered.

A geraniol treatment drastically improved glucose tolerance during OGTT in the *db/db* mice, but the effect was attenuated in non-diabetic C57BL/6 mice and was ablated without glucose gavage. These results may indicate that the glucose regulatory effect of a geraniol treatment is depends on the circulating blood glucose levels.

GLP-1 is expressed not only in the intestinal L cells, but also in the pancreatic α cells, and in the nucleus of the solitary tract (NTS) in the hindbrain region^32^. In particular, a recently published paper revealed that it is pancreatic GLP-1 that contributes to the glucose homeostasis rather than the intestinal source of GLP-1^33^. Considering the dramatic glucose regulatory effects of geraniol treatment, it would be of interest to study the GLP-1 stimulating effects of odorants in the pancreatic islets. In addition, in terms of the initial odorant sensing is occurred in the olfactory epithelium, it would also be of interest that to study the correlation between the olfactory sensations and the roles of the central GLP-1. Given that the GLP-1 is an anorectic gut peptide, to investigate the role of geraniol on the central GLP-1-mediated food intake control would also be interesting.

Odorants distinguish flavor in food as well as tastants. According to our findings, L cells in the gut sense and respond to not only the nutrient content of foods but also to the non-nutrient odor compounds and participate in the gut peptide secretion event, which in turn, affects glucose homeostasis.

## Methods

All experiments were performed in accordance with relevant guidelines and regulation.

### Chemicals

All odorants, DB, metformin, and thapsigargin were purchased from Sigma-Aldrich (St. Louis, MO, USA). Exendin 9-39 (Ex9) was purchased from Abcam (Cambridge, MA, USA). SQ22536 was purchased from Santa Cruz Biotechnology (Santa Cruz, CA, USA).

### Animals

Seven to twelve-weeks old male *db/db* mice and 8-weeks old C57BL/6 mice were purchased from Daehan Biolink (DBL, Eumseong-gun, Chungcheongbuk-do, South Korea). All animal study protocols were approved by the Institutional Animal Care and Use Committee (IACUC) of Kyung Hee University (confirmation number: KHUASP(SE)-14-046).

### *In vitro* GLP-1 secretion assay

Human NCI-H716 cells (74^th^ passage number) were obtained from the Korean Cell Line Bank (KCLB^®^, Seoul, South Korea.) and maintained in RPMI 1640 (Lonza, Walersville, MD, USA) supplemented with 10% fetal bovine serum (FBS; Lonza). Five hundred thousand cells, which the passage number between 78^th^ to 84^th^, were plated in each well of matrigel (Corning, NY, USA, Cat.# 354234)-precoated 24-well plates and maintained for at least 48 h. This procedure, so-called enteroendocrine differentiation, enables the NCI-H716 cells exhibits enteroendocrine cell characteristics including chromogranin and proglucagon expressions as well as GLP-1 secretion^7,8,15,16^. For the odorant screening study, each compound was dissolved in DMSO (1% of total volume) due to the different solubility. Each cell culture plate was separately treated with one dose of one odorant and then tightly covered using paraffin film. After incubation for 1 h in a CO_2_ incubator, GLP-1 concentration was measured using the active GLP-1 ELISA kit (EMD Millipore, Billerica, MA, USA, Cat.# EGLP-35K) according to the manufacturer’s instructions. The active GLP-1 concentrations in each sample were measured using a Fluoroskan Ascent FL machine (Thermo Fisher Scientific, Vantaa, Finland). The lowest level of GLP-1 that can be detected by the GLP-1 assay is 2 pM. *in vitro* studies were performed at least twice using different passages of NCI-H716 cells. The active-GLP-1 levels from each odorant-treated NCI-H716 cells were normalized by the total protein concentration of the corresponding cells and then indicated as fold to the basal. For the basal level GLP-1 secretion, 1 mM CaCl_2_ solution containing 1% DMSO was used as a vehicle. The basal active-GLP-1 secretion levels of the vehicle-treated NCI-H716 cells were ranging from 50 to 70 pM.

### *ex vivo* GLP-1 assay

Eight-week old male C57BL/6 mice were sacrificed to obtain intestinal tissues from the duodenum, jejunum, and ileum as previously described^7^. Dissected mouse intestinal tissues (1 cm) were placed in 12-well tissue culture plates containing DMEM and incubated in 5% CO_2_ incubator for 1 h. And then, the tissue culture medium was replaced with geraniol (100 μM) or glucose (10% of media volume, which is equivalent to 100 mg/ml) containing DMEM. Tissue culture medium was collected after 1 h, and the total GLP-1 concentrations were assayed by GLP-1 multiplex assay kit (Bio-Rad) according to the manufacturer’s instructions. The GLP-1 concentrations in each sample were measured using a Bio-Plex MAGPIX multiplex reader (Bio-Rad), and the results were analyzed with Bio-Plex Manager software (Bio-Rad).

### cAMP ELISA

Endocrine-differentiated NCI-H716 cells were incubated with geraniol. The geraniol-treated cells were collected at 15 min intervals. And the cells were treated with various doses of geraniol for 30 min with or without AC inhibitor, SQ22536. SQ22536 were pre-treated (30 min) to the geraniol treatment. The cAMP concentration was measured as previously described^8^. Briefly, collected cells were lysed using 0.1 M HCl, and the intracellular cAMP was assayed using ELISA (Enzo Life Science, Farmingdale, NY, USA). The results were normalized to the protein concentration of each well.

### siRNA transfection

siRNA duplexes for *OR1A1*, *OR1G1, GNAL, CNGA2, and GNAT3* were synthesized by Bioneer (Bioneer Co., Daejeon, South Korea). The information for each siRNA was provided online (Supplementary Table S1)^34^. A scrambled negative control siRNA was purchased from Bioneer. The negative control siRNA did not affect the *OR1A1*, *OR1G1*, *GNAL*, *CNGA2*, and *GNAT3* expression levels in differentiated NCI-H716 cells. Endocrine-differentiated NCI-H716 cells were transfected with the siRNA duplexes using Lipofectamine RNAiMAX reagent (Invitrogen, Carlsbad, CA, USA) as previously described^9^.

### Real-time quantitative PCR

The expression of ORs and their downstream molecules after the siRNA transfection was determined using a StepOne real-time PCR instrument (Applied Biosystems, Foster City, CA, USA). Total RNA was isolated from the cells, and was then reverse transcribed to cDNA as previously described^8,35^. The expression levels of *OR1A1*, *OR1G1*, *GNAL*, *CNGA2*, and *GNAT3* in each type of siRNA-transfected cell were compared with the corresponding levels in the negative control siRNA-transfected cells, and the 2^−ΔCt^ values were determined^36,37^. *GAPDH* was used as an endogenous control. The sense and antisense sequences of each primer were designed using the Primer express 3.0 software (Applied Biosystems) and were provided online (Supplementary Table S2).

### Taqman quantitative PCR

C57BL/6 mice were sacrificed to obtain duodenum, jejunum, ileum, and OE tissues. Total RNA was isolated from the tissues, and was then reverse transcribed to cDNA as described^8^. The expression levels of *Gnal* and *Olfr43* in each tissue were determined and compared using a StepOne real-time PCR instrument (Applied Biosystems) with Taqman^®^ universal master mix II (Applied Biosystems). *Gapdh* was used as an endogenous control. Taqman probes for the mouse genes *Gnal* (Assay ID: Mm01258217_m1), *Olfr43* (Assay ID: Mm00847191_sH), and *Gapdh* (Assay ID: Mm99999915_g1) were purchased from Applied Biosystems.

The expression levels of *GNAL*, *OR1A1*, and *OR1G1* in the NCI-H716 cells were compared with the *GAPDH* expression using real-time PCR. The Taqman probes for the human genes *GNAL* (Assay ID: Hs00181836_m1), *OR1A1* (Assay ID: Hs01562935_s1), *OR1G1* (Assay ID: Hs00243418_s1), and *GAPDH* (Assay ID: Hs02758991_g1) were purchased from Applied Biosystems.

### Cell viability assay

A cell viability assay was performed using 3-(4,5-dimethylthiazol-2-yl)-2,5-diphenyltetrazolium bromide (MTT; Invitrogen) according to the manufacturer’s instructions^38^.

### Extracellular calcium influx assay

NCI-H716 cells were seeded on a clear-bottom 96-well black plate (Corning, Tewksbury, MA, USA). After differentiation, the medium was replaced with PBS and the mixture was incubated for 30 min with fura-2 AM dye as previously described^8,39^. After 30 min, the medium was replaced with thapsigargin (10 μM) containing PBS with or without 1 mM CaCl_2_ and incubated for a further 30 min. Intracellular calcium levels ([Ca^2+^]_i_) were observed with a Nikon Eclipse TS 100 fluorescence imaging system (Nikon, Melville, NY, USA), and quantified and visualized with InCyt Im2 software (University of Cincinnati, Cincinnati, OH, USA). The number of cells observed was 10–20 per well.

### OGTT

OGTT was performed as previously described^8^. *db/db* mice were overnight fasted (18 h) before the OGTT. Each mouse group was orally administered saline, metformin (2.3 mmol/kg) or geraniol (1, and 2 mmol/kg) just before glucose gavage (5 g/kg). The blood glucose was measured from the tail vein using an Accu-Chek Performa system (Roche Diagnostics, Mannheim, Germany) at 6 time points: 0 (before glucose gavage), 10 (after glucose gavage), 20, 40, 90, and 120 min. The experiments were repeated with the other groups of *db/db* mice, which were pre-injected Ex9 (50 μg/100 ul/mouse) 15 min before the glucose gavage. All *in vivo* studies were performed at least twice using the other sets of mice.

### Plasma GLP-1 and plasma insulin assay

To prevent hypotensive shock, all mice were allowed to rest for one week from the OGTT. Mice were overnight fasted (18 h) before the experiments. Each mouse group was orally administered saline, metformin (2.3 mmol/kg), or geraniol (1- and 2 mmol/kg) just before the glucose gavage (2 g/kg). Fifty-microliters of total mouse bloods were collected from the tail vein using the EDTA-coated 1.5 ml tubes containing DPP-4 inhibitor (EMD Millipore) and protease inhibitor cocktail (Roche). Bloods were collected from the tail vein at 4 time points: 0 (before glucose gavage), 10 (after glucose gavage), and 20 min. The total GLP-1 and insulin concentrations in the mouse plasma were measured as previously described^8^. The same experiments were performed using the Ex9-injected *db/db* mice. A multiplex assay (Mouse Diabetes panel: GLP-1 and insulin; Bio-Rad) was performed as described in the manufacturer’s instructions.

### Statistical analysis

GraphPad Prism 6 software (GraphPad Software, San Diego, CA, USA) was used for the statistical analysis of the experimental results. The statistical significance of each bar chart was measured using one-way ANOVA with Dunnett’s post-hoc (for *in vitro* studies) or Tukey’s post-hoc (for *in vivo* studies). For *in vivo* studies, two-way ANOVA with Tukey’s post hoc (to compare more than 3 groups) or Bonferroni’s post hoc (two compare 2 groups) were performed. P values under 0.05 were considered as significant.

## Electronic supplementary material

Supplementary Information
