# Supplementary material for: Activation of intestinal olfactory receptor stimulates glucagon-like peptide-1 secretion in enteroendocrine cells and attenuates hyperglycemia in type 2 diabetic mice
Source: Sci Rep. 2017 Oct 25;7:13978. doi: 10.1038/s41598-017-14086-5 (PMC5656655; doi:10.1038/s41598-017-14086-5)
Supplement: Supplementary file 1 — Supplementary Information [file 41598_2017_14086_MOESM1_ESM.pdf]

Manuscript ID: SREP-17-05982A

Activation of intestinal olfactory receptor stimulates glucagon-like peptide-1 secretion in enteroendocrine cells and attenuates hyperglycemia in type 2 diabetic mice.

Ki-Suk Kim[1], In-Seung Lee[1], Kang-Hoon Kim[1], Jiyoung Park[1], Yumi Kim[1], Jeong-Hee Choi[2], Jin-Sung Choi[2], and Hyeung-Jin Jang[1,*]

[1]Department of Biochemistry, College of Korean Medicine, Kyung Hee University, 26, Kyungheedae-ro, Dongdaemun-gu, Seoul, 02447, Republic of Korea

[2]College of Pharmacy, The Catholic University of Korea, Bucheon, Gyeonggi-do, 14662, South Korea

[*]Correspondence and requests for materials should be addressed to H.-J.J. (email: hjjang@khu.ac.kr)

Supplementary Table S1. Sequence information for siRNA duplexes.

Gene	Forward	Reverse
OR1A1	CUGAUGUUCGCCUUCACAA (dTdT)	UUGUGAAGGCGAACAUCAG (dTdT)
OR1G1	CUCCUACUCAGGGUGUCUA (dTdT)	UAGACACCCUGAGUAGGAG (dTdT)
GNAL	CGGACCAUUUCUAUCAUCU (dTdT)	AGAUGAUAGAAAUGGUCCG (dTdT)
CNGA2	GACUUGGGAUAAAUCGUGU (dTdT)	ACACGAUUUAUCCCAAGUC (dTdT)
GNAT3	CGAGUGAAAACGACUGGAA (dTdT)	UUCCAGUCGUUUUCACUCG (dTdT)

The product name of each siRNAs: *OR1A1* siRNA, 118058 duplex; *OR1G1* siRNA, 1108121 duplex; *GNAL* siRNA, 10630000 duplex; *CNGA2* siRNA, 1033221 duplex; *GNAT3* siRNA, 1175215 duplex.

Supplementary Table S2. Sequence information for real-time PCR primers.

Gene	Forward	Reverse
GAPDH	GCCACATCGCTCAGACACC	CCCAATACGACCAAATCCGT
GNAT3	CTATGACATGGTCCTCGTGGAA	GATACTGTTGAACAGGTGAAGGCTT
OR1A1	TCAGCCGCCCACTTCACT	CCAGCAATAAGCCAGATACAAGAC
OR1G1	CCCTGGGCTCTGCATCTTC	AGGGAGTGGAGGGCATTCA
GNAL	CCGGGCTCAAGGGATTCT	AAATTAGCCAAGCATGGTAGCAA
CNGA2	GCTGGTGCTGGATTATGTCTCA	TGCGCAATCGGATGAAGAG

Supplementary Figure and legends

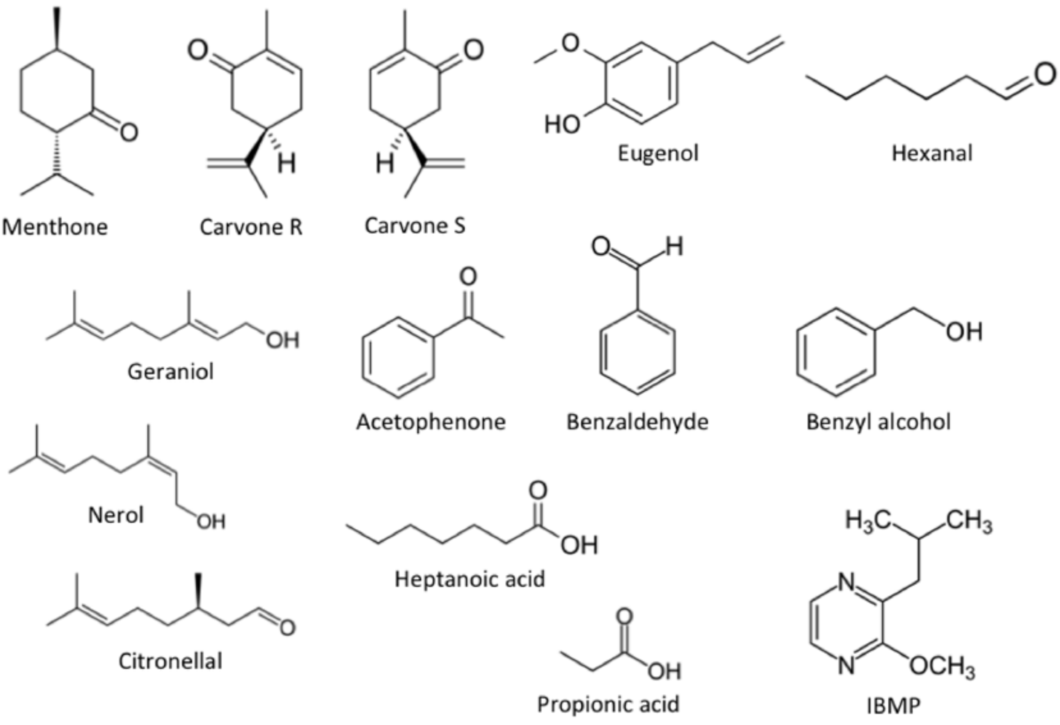

Supplementary Fig. S1. Chemical structure of odorants.

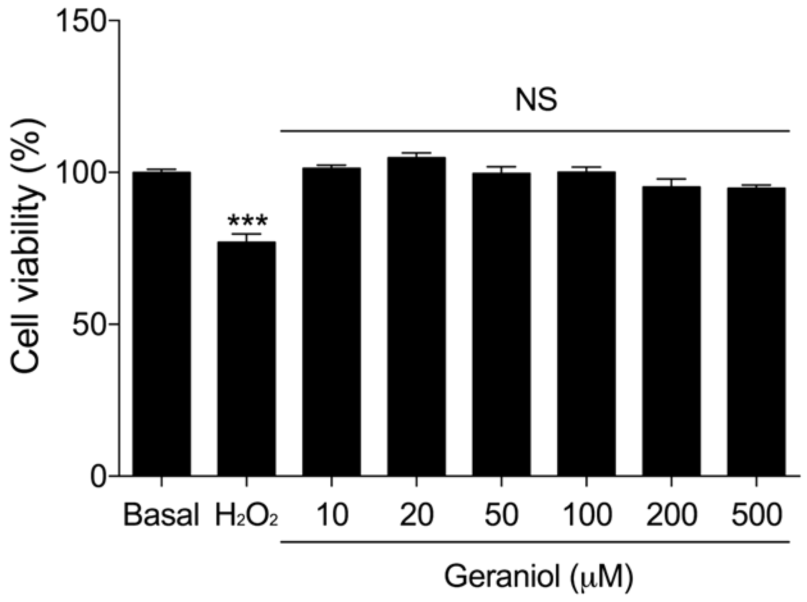

Supplementary Fig. S2. Cell viability assay using geraniol. Error bars show SEM. Statistics, ANOVA followed by Dunnett's post-hoc. ***$p<0.001$. H_2O_2, hydrogen peroxide. NS, non-significant.

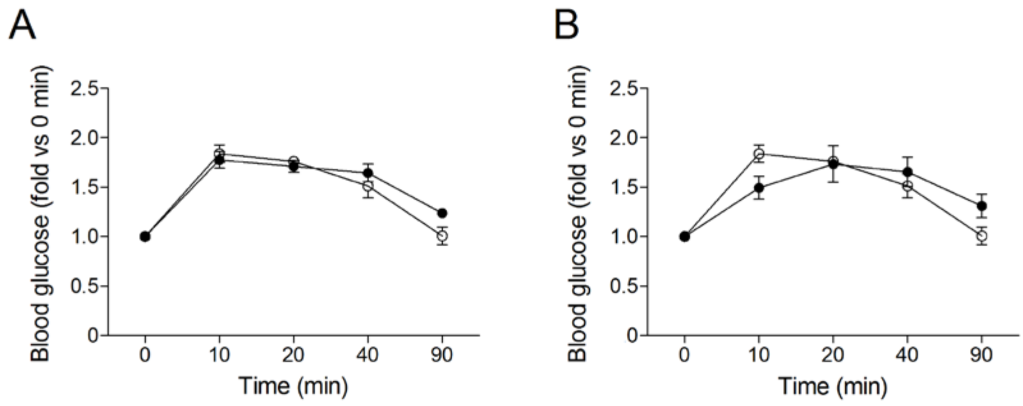

Supplementary Fig. S3. Effects of lower doses of geraniol (a, 2 mmol/kg; b, 0.7 mmol/kg) on blood glucose levels of the *db/db* mice during OGTT (2 g/kg). *n*=4-7. Error bars show SEM.
